# Resilience at the border: traditional botanical knowledge among Macedonians and Albanians living in Gollobordo, Eastern Albania

**DOI:** 10.1186/1746-4269-10-31

**Published:** 2014-03-31

**Authors:** Andrea Pieroni, Kevin Cianfaglione, Anely Nedelcheva, Avni Hajdari, Behxhet Mustafa, Cassandra L Quave

**Affiliations:** 1 University of Gastronomic Sciences, Piazza Vittorio Emanuele 9, I-12042 Pollenzo, (Cuneo), Italy; 2 School of Biosciences and Veterinary Medicine, University of Camerino, Via Pontoni 5, I-62032 Camerino, (Macerata), Italy; 3 Department of Botany, University of Sofia, Blv. Dragan Tzankov, 1164 Sofia, Bulgaria; 4 Department of Biology, University of Prishtina “Hasan Prishtina”, Mother Teresa Str, 10 000 Prishtinë, Republic of Kosovo; 5 Center for the Study of Human Health, Emory University, 550 Asbury Circle, Candler Library 107E, Atlanta, GA 30322, USA; 6 Department of Dermatology, Emory University School of Medicine, 1518 Clifton Rd NE, CNR Bldg. 5000, Atlanta, GA 30322, USA

**Keywords:** Ethnobotany, Albania, Gollobordo, Macedonians, Potato leaves

## Abstract

**Background:**

Ethnobotany in South-Eastern Europe is gaining the interest of several scholars and stakeholders, since it is increasingly considered a key point for the re-evaluation of local bio-cultural heritage. The region of Gollobordo, located in Eastern Albania and bordering the Republic of Macedonia, is of particular interest for conducting ethnobiological studies, since it remained relatively isolated for the larger part of the 20^th^ Century and is traditionally inhabited by a majority of ethnic Macedonians and a minority of Albanians (nowadays both sharing the Muslim faith).

**Methods:**

An ethnobotanical survey focused on local food, medicinal, and veterinary plant uses was conducted with 58 participants using open and semi-structured interviews and via participant observation.

**Results:**

We recorded and identified 115 taxa of vascular plants, which are locally used for food, medicinal, and veterinary purposes (representing 268 total plant reports). The Macedonian Traditional Ecological Knowledge (TEK) was greater than the Albanian TEK, especially in the herbal and ritual domains. This phenomenon may be linked to the long socio-cultural and linguistic isolation of this group during the time when the borders between Albania and the former Yugoslavia were completely closed. Moreover, the unusual current food utilisation of cooked potatoes leaves, still in use nowadays among Macedonians, could represent the side effect of an extreme adaptation that locals underwent over the past century when the introduction of the potato crop made new strategies available for establishing stable settlements around the highest pastures. Additionally, the difference in use of *Helichrysum plicatum*, which is popular in the local Macedonian folk medicine but absent among Albanians, confirms the particular significance of this taxon as it relates to the yellow colour of its flowers in South Slavic folklore.

**Conclusion:**

Botanical studies with an ethnographic approach are crucial for understanding patterns of use of plants within given cultures. Importantly, such studies can also allow for analysis of the dynamics of change in these TEK patterns over the time. The results of this study may be important as baseline data set to be used in rural development programs in Gollobordo, aimed at fostering community-based strategies of management of natural resources.

## Background

Ethnobiological studies conducted in recent years in Eastern Europe have highlighted complex, dynamic systems of folk botanical, mycological, and ecological knowledge [1-28].

This heritage is known in the ethnobiological literature as Traditional Ecological Knowledge (TEK), which has been defined as a "cumulative body of knowledge, practice and belief evolving by adaptive processes and handed down through generations by cultural transmission, about the relationship of living beings (including humans) with one another and with their environment” [29].

In particular, the portion of TEK concerning plants is nowadays increasingly considered crucial in South and South-Eastern Europe for fostering community-based strategies of management of natural resources. It may also represent the starting point for initiatives aimed at the reevaluation of local plants devoted to both small scale food and herbal markets and eco-touristic initiatives [30-37]. Additionally, studies focused on plant uses that have been conducted in Eastern Europe with an in-depth historical or ethno-historical approach [38,39] or via archival research and/or contemporary surveys conducted among botanists remembering their childhood [40-44] have demonstrated how plant perceptions change over time, in response to a complex interplay of socio-cultural, environmental, and economic dynamics.

In the past few years, we have concentrated our research on the botanical knowledge overlaps and exchanges between South Slavs and Albanians in multi-cultural or bordering areas in South-Eastern Europe [26,39] and on the *resilience* of TEK [45] among diasporas in the same area [46]. In these studies, we observed phenomena of hybridization of botanical knowledge, as well as a more “herbophilic” [47] attitude of the Slavs in comparison to the Albanians. In the current study, we wanted to further analyze the local botanical knowledge systems among Macedonians and Albanians living in the Gollobordo region, in Eastern Albania.

We could postulate that ethnic Macedonians in this area in Albania preserved much of their original folk botanical heritage because of their isolation in the past decades. This isolation was especially evident during the Communist period (1945–1991), both from the Albanian neighbors and also from those Macedonians, who remained after the creation of the Albanian state (1912) on the other side of the border (at the time within the territory of the Kingdom of Serbia, later Kingdom of Serbs, Croats and Slovenes and then Yugoslavia, nowadays Republic of Macedonia). Nevertheless, the fact that both the Albanian and Macedonian communities of Gollobordo share the same faith (Islam) for the most part, with some intermarriage in the past decades, there may have been some exchange of botanical knowledge. In order to assess all of this, we designed the objectives of this study to: 1) record traditional uses of local botanicals (both cultivated and wild) for food, medicine, and veterinary purposes among Macedonians and Albanians; 2) verify the occurrence of an expected richer, “more conservative” Macedonian ethnobotany; and 3) analyze differences and commonalities in the traditional plant knowledge between the two communities and to propose some explanatory models.

## Methods

### Study area

**Figure 1:**
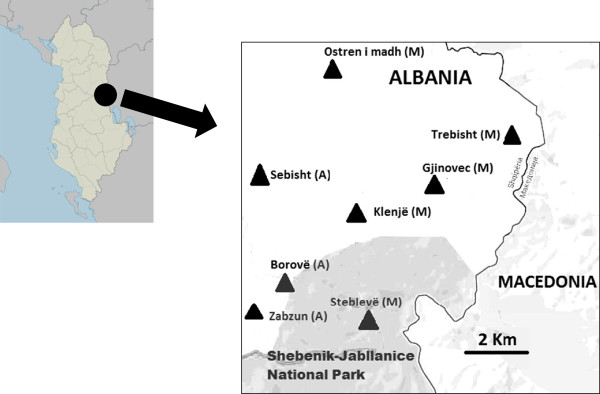
The study area.

The current study was conducted in eight villages of the mountain of the Gollobordo area, in Eastern Albania, bordering the Republic of Macedonia (Figure 1); the focus was on three villages inhabited by ethnic Macedonians: Klenje (1,203 m.a.s.l), Gjinovec (1,252 m.a.s.l.), and Steblevë (1,200 m.a.s.l.) – this last village included within the newly established Shebenik–Jabllanice National Park, with an overall permanent population of approx. 300 inhabitants (while Gjinovec is only inhabited nowadays during the late spring and summer months); and three villages inhabited by Albanians: Sebisht (915 m.a.s.l.), Borovë (940 m.a.s.l.), and Zabzun (1,028 m.a.s.l.), with an overall permanent population of approx. 300 inhabitants as well. Additionally, in order to have a sample more adherent to the ethnic proportion of Gollobordo (for which more than two-thirds is inhabited by Macedonians), a few additional interviews were also conducted in the larger Macedonian villages of Ostren i madh (948 m.a.s.l., approx. 1000 inhabitants) and Trebisht (782 m.a.s.l., approx. 1,000 inhabitants).

**Figure 2:**
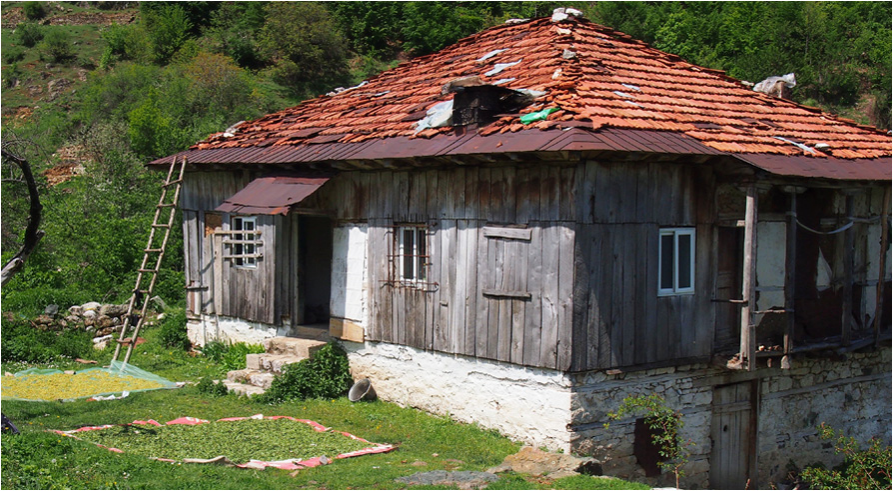
Typical Macedonian house inhabited nowadays only in the late spring and summer season in Gjinovec (1,252 m.a.s.l.).

The local economy is based on small-scale farming and pastoralist activities, with a significant portion of the population that migrates to Tirana and/or other city centers and sometimes back for a few months in their villages only during the late spring and summer months (Figure 2). According to the Albanian Institute of Statistics data, Gollobordo and the entire Eastern and North-Eastern region of Albania (covering Peshkopia and Kukës counties) are among the economically poorest areas of not only the country, but also all of Europe [48]. All of the villages in the Gollobordo are nowadays permanently inhabited only by families of Islamic faith, while until the 1990s, most of the Macedonian villages also had an important Christian Orthodox component. The local dialect of the Macedonian minority, now spoken by less than 3,000 inhabitants, has been the focus of a number of studies conducted by Slavic linguists in the past Century. Two remarkable field ethnolinguistic and ethnographic studies have also been conducted in Gollobordo in recent years [49,50].

The climate of this area is continental, with very harsh temperatures and snowfall during the winter season. The landscape around these villages is dominated by low mountains covered by the *Quercus frainetto* woodland belt, and by the *Fagus sylvatica* woodland belt at higher elevations. Sometimes it is possible to find some woodland fragments of *Quercus cerris* (in soil containing more clay) and *Castanea sativa* (in more acidic soil); in addition, there is some reforestation by *Pinus nigra*, probably carried out during the Communist period (1945–1991). The landscape is also covered by large extensions of secondary patches of semi-natural dry and humid grassland. A riparian marshy vegetation is found along the valleys, which is frequently fragmented and residual, dominated by some species of *Salix*, such as *S. alba, S. eleagnos* (sometimes really large) and, less frequently, *S. purpurea*. In the secondary succession, it is easy to find some different shrub species such as *Corylus avellana, Cornus mas, Juniperus communis, Crataegus monogyna, Crataegus sericea* and *Juniperus oxycedrus*. Up to the village of Klenje, within a high plateau, we could observe a large population of *Prunus cocomilia*.

The main herbal vegetation in the villages is anthropogenic, with ruderal/nitrophylic species and cultivars like *Vitis labrusca*, some fruit trees (esp. *Prunus* species) and some vegetables; *Vitis labrusca* is traditionally cultivated climbing on trees with light pruning.

The main trees are situated in a gradient between wild and domesticated conditions: *Fraxinus excelsior, Quercus cerris, Q. frainetto* (mostly as totem trees), *Prunus avium, P. domestica, P. cerasus, P. cerasifera, Juglans nigra, Cydonia oblonga, Malus domestica, Pyrus communis*, *Robinia pseudoacacia, Populus nigra*, *Ailanthus altissima*, *Syringa vulgaris*, *Acer campestre*, while the most common shrubs are *Rosa canina* s.l. and *Rubus hirtus, R. caesius, R. ulmifolius,* and *Clematis vitalba*.

### Field study

In May 2013, in-depth open and semi-structured interviews were conducted with community members (n = 58, 43 Macedonians and 15 Albanians; age between 9 and 87 years old), which were selected using snowball sampling techniques. Study participants were asked about traditional uses of food, medicinal, veterinary, and ritual plants (in use until a few decades ago or still in use nowadays) via semi-structured and open interviews, walks in the natural environment in the proximity of the villages together with informants, and participant observation within the households. Specifically, local name(s) of each quoted taxon, the plant part(s) used, in-depth details about its/their manipulation/preparation and actual medicinal or food use(s) were recorded. Interviews were conducted in Albanian or Macedonian languages with the help a bilingual simultaneous translator. Prior informed consent was always verbally obtained prior to conducting interviews and researchers adhered to the ethical guidelines of the American Anthropological Association [51]. During the interviews, informants were always asked to show the quoted plants. Voucher specimens were taken for the wild taxa, when available, and are deposited at the herbarium of the School of Biosciences and Veterinary Medicine of the University of Camerino, Italy (*Herbarium Universitatis Camerinensis*; acronym: CAME).

Taxonomic identification was conducted according to the official Flora of Albania [52-55] and the previous Albanian Excursion Flora [56]. For *Crataegus* spp. we referred to the Rosaceae’s taxonomy in Euro + Med PlantBase [57]. Family assignations follow the Angiosperm Phylogeny Group III system [58]. Local plant names were transcribed following the rules of the standard Ghegh Albanian and Macedonian languages.

## Results and discussion

Overall, we recorded the local uses of 116 taxa of vascular plants; we documented 268 plant reports, 105 for food, 87 for human medicine, and 76 for veterinary applications.

Given the variety of means through which data were elicited in the field, detailed cultural importance, frequency and consensus indexes, which do always require consistently performed interviews, were not considered in the data analysis.

However, in the tables and in the data used for the comparison we included only plant uses reported by at least two informants, as well as those uses, which were quoted by the majority of the interviewees.

### Food plants

The food use of 55 taxa were recorded, 25 of which are wild or semi-domesticated (Table 1).

**Table 1:** Local food plant uses recorded in the study area

**Taxon, family and voucher specimen code**	**Recorded Albanian folk name(s)**	**Recorded Macedonian folk name(s)**	**Status**	**Plant part(s) used**	**Traditional modalities of consumption and other recorded information**	**Alb**	**Mac**
*Allium cepa* L.	**Qepa**	**Кромид**	C	AP; Bu	Raw and cooked (traditionally filling for pies made with corn flour - *byrek*^ALB^/*komat*^*M*AC^)	+	+
Amaryllidaceae							
*Allium porrum* L.	**Pras**	**Праз**	C	AP	Filling for pies	+	
Amaryllidaceae							
*Allium sativum* L.	**Hurdhëra**	**Лук**	C	Bu	Seasoning	+	+
Amaryllidaceae							
*Atriplex hortensis* L.	Laboda	Лабода, Лобода	C	L	Filling for pies	+	+
Amaranthaceae							
*Beta vulgaris* L.	Panxhari		C	R	Ingredient for making halva*	+	
Amaranthaceae							
				L	Filling for pies		+
*Brassica oleracea* L.	**Liakër, Liakra**	**Зелка, Расол**	C	L	Pickled/lacto-fermented in water and salt); the liquid resulting from of the lacto-fermentation (*rasol*) was eaten with bread by the poorest community members*	+	+
Brassicaceae							
					*Sarma*	+	
				L	Filling for pies		+
*Capsicum annuum* L.	**Spec**	**Пиперка**	C	Fr	Cooked	+	
Solanaceae							
					Lacto-fermented in water and salt or in yogurt ricotta	+	+
*Castanea sativa* Mill.	Kostenja		W	Fr	Boiled or roasted	+	+
Fagaceae							
CAME 26314							
*Cornus mas* L.	**Thana**	**Дрен**	W	Fr	Fermented and distilled into *raki*	+	+
Cornaceae							
CAME 26279							
					Fermented into vinegar	+	+
					Syrup and compote (dried fruits boiled with water and sugar)	+	+
					Concentrated syrup/soft jam (*pekmez*)	+	
					Jam		+
					(Fermented?) beverage obtained boiling the fruits in water for a few hours (the resulting beverage is kind of a fruit soda, which is kept in the fridge or in the cellar; considered very healthy, is often consumed adding sugar)		+
*Corylus avellana* L.	Leithija	Лешник	W	Se	Raw and dried	+	+
Betulaceae							
CAME 26242							
*Crataegus monogyna* Jacq.	**Cut, Murriz**	**Глогиня, Глогиня дива** (*C. monogyna*), **Глогиня питома** (*C. sericea*)	W	Fr	Snack		+
Rosaceae							
CAME 26280							
*Crataegus sericea* Dzekov ^§^							
Rosaceae							
CAME 26278							
					Fermented beverage		+
					Fermented into raki		+
*Cucumis sativus* L.	**Kastravec**	**Kраставицa**	C	Fr	Pickled/lacto-fermented (in water and salt)	+	+
Cucurbitaceae							
CAME 26291							
*Cucurbita maxima* Duch	Kungull	Tиква	C	Fr	Filling for pies; pickled/lacto-fermented (in water and salt)	+	+
Cucurbitaceae							
*Cydonia oblonga* Mill.	Ftoi	Дуња	C	Fr	Jams (sometimes prepared dipping in a preliminary procedure the fruits pieces in water and lime, then boiling with sugar, so that the fruit pieces remained hard at the end)	+	+
Rosaceae							
CAME 26290							
					Compote (fruits boiled with water)		+
					Fermented (?) beverage obtained boiling the fruits in water for a few hours (the resulting beverage is kind of fruit soda, which is kept in the fridge or in the cellar		+
*Daucus carota* L.			C	R	Lacto-fermented in water and salt	+	
Apiaceae							
CAME 26208							
*Fagus sylvatica* L.	**Ahu**	**Бук**	W	K	Raw as snack (however, consumption of large amounts may generate headaches)*	+	+
Fagaceae							
CAME 26249							
				Wo	Fuel for smoking meat	+	
*Fragaria × ananassa* Duch. ex Rozier	Lule shtrydhe		C	Fr	Raw	+	
Rosaceae							
*Fragaria vesca* L.	**Derthea, Dirthene**	**Ягодка, Ягода**	W	Fr	Raw	+	+
Rosaceae							
CAME 26247							
*Helianthus tuberosus* L.		Шалгун	SD	T	Raw or cooked		+
Asteraceae							
CAME 26312							
*Hordeum vulgare* L.	Elb	Jaчмен	C	Frfl	Bread (mixed with rye flour)*	+	
Poaceae							
				Fr	Roasted and decocted, as a kind of coffee	+	
*Juglans regia* L.	**Arra**	**Орев**	SD; C	K	Raw, or various cakes	+	+
Juglandaceae							
CAME 26238							
*Juniperus communis* L.	Dllinja	Смрека	W	Fr	Fermented and distilled into *raki*	+	+
Cupressaceae							
CAME 26253							
					Seasoning ingredient for lacto-fermented vegetables		+
*Lactuca sativa* L.	Sallata	Лоштика	C	L	Raw of filling for pies		+
Asteraceae							
*Malus domestica* Borkh.	**Molla** (Molla e kuqe, Sterkinka)	**Јаболка** (италианска, кисели, Ренета, Штерка)	C	Fr	Raw and dried (*hoshaf*)	+	+
Rosaceae							
CAME 26236							
					Sliced and dried; consumed boiled		+
				Fr	Fermented and distilled in *raki*	+	
*Malus sylvestris* (L.) Mill.	**Molla e egër**	**Дива Јаболка**	W	Fr	Dried (*hoshaf*)		+
Rosaceae							
CAME 26288							
					Fermented into vinegar	+	+
*Medicago sativa* L.	Jonxha		C	L	Cooked, as an emergency (famine) food*	+	
Fabaceae							
CAME 26292							
*Morus alba* L.	Mani	Mурвинка	C	Fr	Jams and compote	+	
Moraceae							
CAME 2631							
					Fermented and distilled into *raki*	+	
*Phaseolus vulgaris* L.	**Fasulja, Grosh**	**Грав**	C	Fr; Se	Cooked	+	+
Fabaceae							
				L	Filling for pies		+
					*Sarma*		+
				UF	Cooked, filling for pies		+
*Prunus cerasifera* Ehrh.	**Kumbullë Elbasani, Kumbullë kokormadhe**	**Слива Елбасанска**	SD	Fr	Fermented and distilled in *raki*	+	+
Rosaceae							
CAME 26266							
					Fermented (?) beverage obtained boiling the fruits in water for a few hours	+	
					Jam		+
*Prunus avium* (L.) L.	**Qershija**	**Цреша (питома)**	C	Fr	Raw	+	+
Rosaceae							
CAME 26240							
*Prunus cerasus* L.							
CAME 26298							
Rosaceae							
					Jams	+	+
					Compote (fruits boiled with water and sugar)		+
					Fermented (?) beverage obtained boiling the fruits in water for a few hours (the resulting beverage is kind of fruit soda, which is kept in the fridge or in the cellar)		+
					Dye for hard boiled eggs to which are consumed on St. George’s Day, as a good omen		+
*Prunus cerasus* var. *marasca* (Host) Vis.	Qershija e egër	Дива цреша	W; C	Fr	Raw	+	+
Rosaceae							
CAME 26299							
*Prunus cocomilia* Ten.		**Дива слива**	W	Fr	Fermented and distilled into *raki*		+
Rosaceae							
CAME 26277							
*Prunus domestica* L.	**Kumbulla**	**Слива** (блага, магарица, жолта, синица)	C	Fr	Raw and dried	+	+
Rosaceae							
CAME 26239							
					Fermented and distilled into *raki*	+	+
					Jam	+	+
					Fermented (?) beverage obtained boiling the fruits in water for a few hours (the resulting beverage is kind of fruit soda, which is kept in the fridge or in the cellar)		+
*Prunus spinosa* L.	Kolumbria, Kurmulia	Дива слива	W	Fr	Gathered after the frost and consumed raw as snack, or fermented and distilled into *raki*, or transformed into a compote	+	
Rosaceae							
CAME 26260							
*Pyrus communis* L.	**Dardha**	**Kруша**	C	Fr	Raw and dried	+	
Rosaceae							
CAME 26306							
					Compote (fruits boiled with water and sugar)		+
					Jam	+	
					Fermented and distilled into *raki*	+	
					Fermented (?) beverage obtained boiling the fruits in water for a few hours (the resulting beverage is kind of fruit soda, which is kept in the fridge or in the cellar)		+
*Pyrus pyraster* (L.) Burgsd.	**Dhardhë e egër, Gorrica**	**Дива круша**	W	Fr	Gathered after the frost, ripened on straw, and consumed dried or in compote	+	+
Rosaceae							
CAME 26244							
*Pyrus amygdaliformis* Vill.							
Rosaceae							
CAME 26316							
					Jam		+
*Ribes multiflorum* Kit. ex Roem. et Schult.		Диво грозje	W	Fr	Snack		+
Grossulariaceae							
CAME 26263							
*Rubus idaeus* L.		Малина	W; C	Fr	Snack		+
Rosaceae							
CAM 26321							
					Syrup and compote (fruits boiled with water)		+
*Rubus hirtus* Waldst. et Kit.	**Fermoza, Manaferra**	**Капина**	W	Fr	Raw and jams	+	+
Rosaceae							
CAME 26258							
*Rubus ulmifolius* Schott							
Rosaceae							
CAME 26310							
*Rubus caesius* L.							
Rosaceae							
CAME 26245							
					Syrup and compote (fruits boiled with water)		+
					Fermented and distilled in *raki*	+	
*Rumex acetosa* L.	Ufull, Uthull	Киселец	W	L	Boiled, then in filling for pies (*byrek*^ALB^/*komat*^MAC^) or as vegetables cooked with rice and dairy products (*buranje*^ALB^*/zelje*^MAC^); traditionally dried and then used during the whole winter		+
Polygonaceae							
CAME 26243							
*Rumex conglomeratus*							
Murray							
CAME 26286							
Polygonaceae							
					Infusion: to be used for preparing yogurt (if starter culture is missing)		+
				L; St	Snack	+	+
*Rumex patientia* L.	**Lepjeta**	**Щавел**	W	L	Boiled, then used filling for pies or as vegetables cooked with dairy products; traditionally dried and then used during the winter	+	+
Polygonaceae							
CAME 26285							
*Secale cereale* L.	**Thekna**	**Рж**	C	Frfl	Mixed with corn flour: bread, esp. in the past*	+	+
Poaceae							
				Fr	Roasted and decocted, as a kind of coffee	+	
*Solanum lycopersicum* L.	**Domate, Patlixhan kuqe**	**Црвени патлиџани**	C	UF	Lacto-fermented in water and salt	+	+
Solanaceae							
				Fr	Raw and cooked	+	+
				L	Cooked (emergency/famine food)*	+	
*Solanum melongena* L.	Patlixhan i zezë	Црни патлиџани	C	Fr	Cooked	+	
Solanaceae							
*Solanum tuberosum* L.	**Patate**	**Компири**	C	T	Cooked	+	+
Solanaceae							
				YL	Filling for pies (only in the past among Albanians)*. The bitter taste is particularly appreciated by the Macedonian communities; young potatoe leaves are the most common filling for the traditional pie (*komat*) in June, after the young nettle’s season end	+	+
					*Sarma*		+
*Taraxacum officinale* Weber ex F.H. Wigg.	Qumështore	Млечак, Млекаица, Жело	W	L	Salads		+
Asteraceae							
CAME 26289							
*Urtica dioica* L.	**Hjeth, Hisel, Hithra, Hith**	**Коприва**	W	YL	Boiled, then used in filling for pies or cooked with rice and dairy products	+	+
Urticaceae							
CAME 26262							
*Vaccinium myrtillus* L.	**Borovnica, Rrush i egër**	**Диво грозje, Цршине**	W	Fr	Snack	+	+
Ericaceae							
					Fermented into a fruit soda-like beverage		+
					Compote		+
*Vitis labrusca* L*.*	**Rrush** (variety Çelek)	**Грозje** (Шилек)	C	Fr	Fermented into wine or vinegar	+	+
Vitaceae							
CAME 26265							
					Concentrated juice (*pekmez*)		+
					Fermented (?) beverage obtained boiling the fruits in water for a few hours (the resulting beverage is kind of fruit soda, which is kept in the fridge or in the cellar		+
				L	*Sarma*	+	+
*Zea mays* L.	**Misër**	**Пченка**	C	Frfl	Bread, pies	+	+
Poaceae							
				YL	Filling for pies		+
Diverse tree species			W; C	WA	Added to flour, water, and eggs for producing home-made noodles (*jufka*)	+	
					As a disinfectant, rubbed onto the sheep’s stomach before it is cooked and eaten		+
					Added to water when boiling corn		+

^§^: first record of the species in Albania.

In bold: folk taxa quoted by more than 40% of the informants.

C: cultivated; SD: semi-domesticated; W: wild.

*: past use.

+: recorded use.

Plant part(s) used: *AP* aerial parts; *Bu* bulbs; *Fr* fruits; *Frfl* flour from fruits; *K* kernels; *L* leaves; *R* roots; *Se* seeds; *St* stems; *T* tubers; *UF* unripe fruits; *Wo* wood; *WA* ashes from wood; *YL* young leaves.

Among the most uncommon uses, we have to mention the use of potato leaves, both for *sarma* (leaves rolled around a minced meat and rice filling) and especially as filling for white corn-flour based pies (*laknur* or *byrek* in Albanian, *komat* in Macedonian), which is still very common among the Macedonians living in the highest villages of Gollobordo, while among Albanians this was remembered as a past use only. We found this use of potato leaves as filling for savory pies to be quite common in Gollobordo in June, after the “nettle season” (*Urtica dioica*), which is the primary wild plant used in the early spring, while *Rumex* spp. dominates later in the season as a pie filling ingredient. We recently found a similar relictual use among the last Albanians living in the upper Reka valley, on the Macedonian side of Mount Korab [39].

The archaeologist Michael Galaty and his team have recently conducted intensive field research in the mountainous Shala Valley in Northern Albania. Galaty has proposed that the Little Ice Age and the introduction of maize, which took place in the Balkans starting from the 16^th^ Century [59,60], played a crucial role in the remarkable demographic expansion in this area in the 17^th^ and 18^th^ Century [61]. We believe that the introduction of the potato crop (*Solanum tuberosum*) in the mountainous areas of the Western Balkans and in the Gollobordo area (presumably at the end of the 19^th^ Century) may have also similarly determined a remarkable vertical expansion of the inhabited landscape, offering locals for the first time in the history of the region the possibility to permanently colonize and settle the higher pastures. As a side-effect of this shift, which was sustained by an increase of food resources (dairy products and potatoes), the leaves of the potato plant may have also been considered as a vegetable, especially in the spring, where this would have largely become available and when not many other green leafy vegetables are available (apart from wild nettles and *Rumex* spp.). The toxic glycoalkaloid content of the potato leaves could perhaps be reduced by the way in which they are traditionally collected and prepared. Only the young leaves are gathered and they are boiled in water before being used as a pie filling. Indeed, research on the chemistry of *S. tuberosum* leaves has demonstrated that glycoalkaloid content (measured by levels of α-solanine and α-chaconine) are at their lowest in the young leaves, with those appearing on the most distal-location of the stem having the overall lowest glycoalkaloid content [62]. However, the boiling step likely reduces small level of the overall glycoalkaloid content, thus the final product would be expected to contain a somewhat bitter quality, and indeed, our participants confirmed that the pie made with potato leaves is appreciated exactly because of its “bitter taste”.

On the other hand, the consumption of “bitter” potatoes (with high glycoalkaloid contents) has been well discussed by Timothy Johns [63] for the case of the Aymara population in Southern America, where bitter potato varieties (*jank’o* and *luq’i*) were often eaten unprocessed after the harvest.

Other important uncommon cultivated food sources we found included the young leaves of corn (*Zea mays*) as pie filling, and bean leaves for use in *sarma.* Upon consideration of trees, the rare food use of *Prunus cocomilia* for producing home-made *raki* should be better analyzed under the viewpoint of sensory analysis for possible local economic development outcomes. In fact, the local know-how on mixing, fermenting, and home-distilling various *Prunus* tree fruits in Gollobordo, as in other areas of the Balkans, seems to be extremely sophisticated.

### Medicinal plants

**Figure 3:**
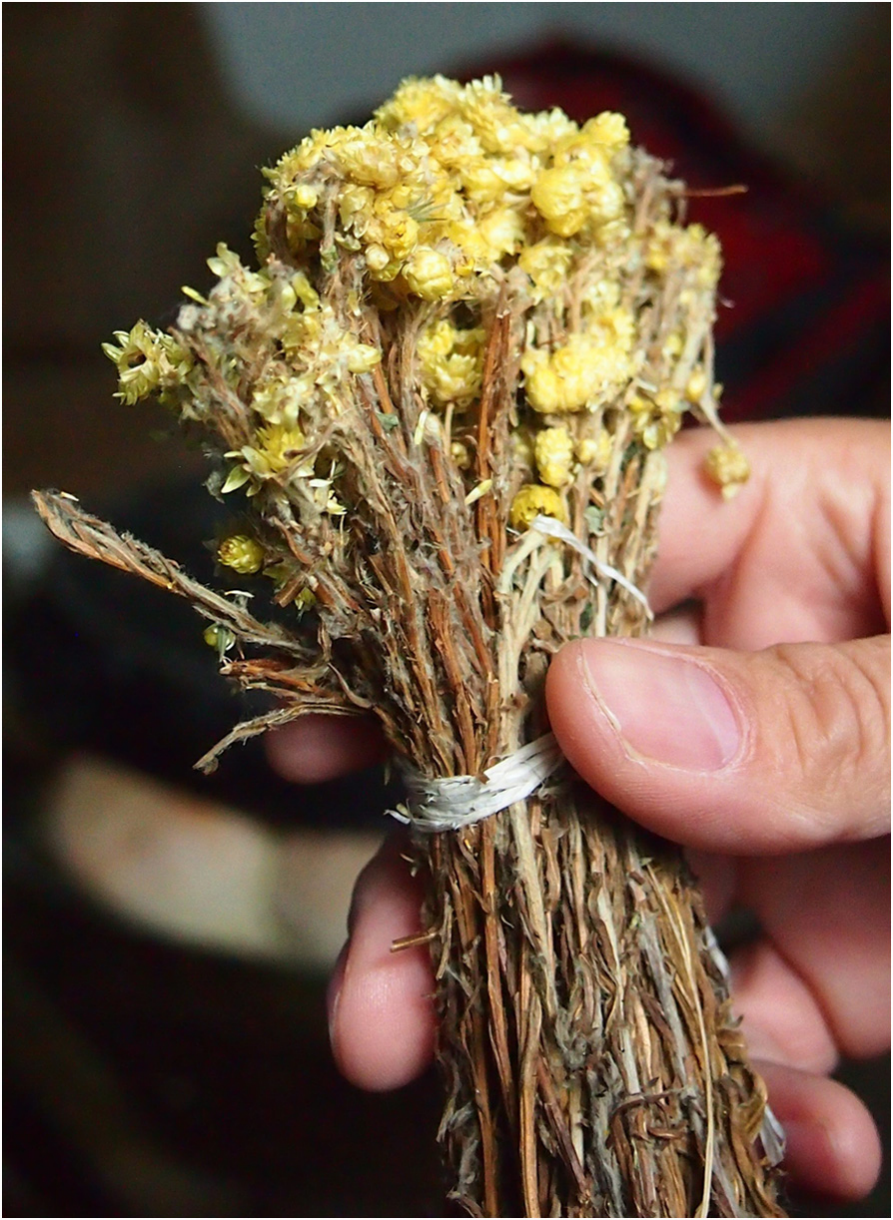
**Dried flowering aerial parts of *Helichrysum *** **sp.**

The recorded local uses of 53 medicinal plant taxa are reported in Table 2. It is worthwhile to mention the case of *Helichrysum plicatum* (Figure 3), which is the most quoted taxon among the Macedonians of Gollobordo. Within this ethnic group, this medicinal herb is the most frequently used remedy as it is applied in the treatment of many diseases as a kind of panacea. The high cultural consensus concerning the use of *Helichrysum* spp. in the Macedonian and Bulgarian medical folklore is remarkable in the scientific literature. A number of folk names referred to this taxon in Bulgarian retain the root “smil”, which has the meaning of physical beauty and health; moreover, in Bulgarian folk medicine, this taxon has been considered to be a real panacea and is often used for many purposes: as a diuretic, against dropsy, liver diseases, jaundice, stagnation of blood in the abdomen, tinnitus, low blood pressure, bone spikes, rheumatism, sciatica, rickets, worms, deafness and for treating skin diseases [64,65]. The ritual use of this plant in the South Slavic folklore is often linked to the bright yellow color of its flowers, which symbolizes sun and light, virginity, moral purity, and mercy in the Balkan folkloric tradition [66]. In Bulgaria, *Helichrysum* had to be collected in the morning of *Georgyovden* (corresponding to St. George’s day, May 6^th^) and were sewn into the hem of garments as an amulet. In order to prevent jaundice in newborns, a bunch of *Helichrysum* was placed under the infant’s pillow. The flowering aerial parts of this plant were used in wedding bouquets and the plant is mentioned in wedding songs and used as a sign of marriage [65,67]. Additionally, flowers of *Helichrysum* were believed to be able to provide a girl with a fiancé; according to this belief, while the flower is fresh, the girl will be a maiden, when it has withered – she will be engaged, and when it is dried – she will marry [65].

**Table 2:** Medicinal local plant uses recorded in the study area

**Taxon, family and voucher specimen code**	**Folk name(s) recorded among Albanians**	**Folk name(s) recorded among Macedonians**	**Status**	**Plant part(s) used**	**Recorded modalities of medicinal uses(s) and treated pathologies**	**Alb**	**Mac**
*Achillea collina* (Becker ex Rchb.f.) Heimerl		Бело цвеkе	W	Fl	Decoction: cicatrizing on wounds		+
Asteraceae							
*Achillea millefolium* L.							
Asteraceae							
CAME 26294							
*Allium cepa* L.	#	#	C	J	Instilled in the ear for treating earaches		+
Amaryllidaceae							
*Allium porrum* L.	#	#	C	AP	Heated, mixed with water and salt, to externally treat chilblains		+
Amaryllidaceae							
				J	Instilled in the ear for treating earaches	+	
*Allium sativum* L.	#	#	C	Bu	Consumed as an anti-hypertensive	+	+
Amaryllidaceae							
					To be worn as amulet against the evil-eye in the or in necklaces (*sysh, naok*)	+	+
*Arum italicum* Mill.	Shakulliza		W	Fr	Infusion: arthritis	+	
Araceae							
*Asplenium trichomanes* L.	Fier guri		W	L	Infusion: diuretic	+	+
Aspleniaceae							
CAME 26293							
*Bovista* sp.	**Fenë arrushe, Fushkaica**	**Пуша**	W	DFB	Externally applied on wounds	+	+
Agaricaceae							
*Cirsium arvense* (L.) Scop.		Овцец	W	AP	Fodder		+
Asteraceae							
*Cornus mas* L.	#	#	W	Fr	Consumed as snack for strengthening the heart		+
Cornaceae							
CAME 26279							
					Fermented into vinegar, applied on the breast: anti-fever	+	
					Fermented and distilled in raki, drunk: anti-asthmatic; believed to able to treat “seven” diseases		+
				FB	As part of the *lule ditvere* (“flower of the summer”) bunch, which is hang in March on the churn and on the stable doors, as a good omen for the diary production	+	+
*Corylus avellana* L.	#	#	W	FB	As part of the *lule ditvere* (“flower of the summer”) bunch, which is hung in March on the churn, as a good omen for the dairy production		+
Betulaceae							
CAME 26242							
*Crataegus monogyna* Jacq.	#	#	W	Th	Externally applied: for treating snake bites	+	
Rosaceae							
CAME 26280							
*Crataegus sericea* Dzekov^§^							
Rosaceae							
CAME 26278							
				L; F	Infusion: for treating headaches, insomnia, hypertension, anti-rheumatic, anti-cancer		+
				Fr	Decoction: cardiotonic, stomachaches, anti-fever, anti-rheumatic		+
				FB	As part of the *lule ditvere* (“flower of the summer”) bunch, which is hung in March on the churn and on the stable doors, as a good omen for the dairy production; same at St. George’s day (May 6^th^ )		+
*Cruciata laevipes* Opiz	Gjak edhe qumësht		W	AP	Crushed, mixed with salt, and given as fodder to the sheep at St. George’s Day (May 6^th^): considered propitiatory for the good health of the animals	+	
Rubiaceae							
CAME 26276							
*Cydonia oblonga* Mill.	#	#	C	L	Infusion: stomachache	+	+
Rosaceae							
CAME 26290							
*Euphorbia characias* L.	**Rrydh, Shpengull**	**Лишај**	W	R	As part of the *lule ditvere* (“flower of the summer”) bunch, which is hang on the churn, as a good omen for the diary production	+	+
Euphorbiaceae							
CAME 2628							
*Fraxinus excelsior* L.		Jасика	W	L	Infusion: diuretic		+
Oleaceae							
CAME 26304							
*Helleborus odorus* Waldst. et Kit. ex Willd.	**Kukurek, Lule ditvere**	**Кукурек**	W	R	Inserted on the horse ear: panacea	+	
Ranunculaceae							
CAME 26282							
				F	As part of the *lule ditvere* (“flower of the summer”) bunch, which is hang on the churn, as a good omen for the dairy production	+	+
*Helichrysum plicatum* DC. and other *Helichrysum* species	Borsillok i verdhë	**Свилен**	W	FAP	Infusion: appetizing, stomachaches, as a digestive, anti-diarrheal, cardiotonic, diuretic, anti-moths		+
Asteraceae							
CAME 26274							
					Infusion: hepatitis	+	
*Hypericum perforatum* L.		**Балсам**	W	FAP	Infusion: stomachache		+
Hypericaceae							
CAME 26270							
*Juglans regia* L.	#	#	SD	UF	Infusion: for treating hyperthyroidism		+
Juglandaceae							
CAME 26238							
					Crushed, externally applied on the hair as dyeing agent	+	+
*Juniperus communis* L.	#	#	W	Fr	Infusion: diuretic, stomach-aches, anti-cold, bechic		+
Cupressaceae							
CAME 26253							
					Fermented and distilled into raki, which is drunk for treating asthma		+
*Juniperus oxycedrus* L.		Смрека	W	Fr	Infusion: bechic	+	
Cupressaceae							
CAME 26267							
*Malus domestica* Borkh.	#	#	C	Fr	Sliced and dried; consumed boiled for treating stomachache		+
Rosaceae							
CAME 26236							
*Malva sylvestris* L.		Леблебија	W	Fr	Snack		+
Malvaceae							
CAME 26295							
*Matricaria chamomilla* L.	Kamomill		W	FT	Infusion: recreational	+	
Asteraceae							
*Melissa officinalis* L.	Bosillek Micël		C	AP	Infusion: headaches		+
Lamiaceae							
CAME 26235							
					Infusion in external washes for newlyweds, as a good omen	+	
					High dosage to be avoided by males, who could lose their libido		+
*Nicotiana tabacum* L.	Duhan		C	L	Dried and ground (tobacco), externally applied on wounds	+	
Solanaceae							
*Orchis* spp.	**Salep**	**Салеп**	W	R	Dried, powdered, then in decoction: panacea, reconstituent (often consumed with bread); to improve fertility in males	+	+
Orchidaceae							
					Dried, powdered, then in decoction: hepatitis		+
*Origanum vulgare* L.	**Bozillek i malit, Çaj i malit, Çaj i zi, Çaj veni**	**Планински чај**	W	FT	Infusion: recreational, anti-flu, bechic	+	+
Lamiaceae							
CAME 26233							


					Infusion: anti-hepatitis	+	
					Infusion: for treating stomachaches, panacea	+	
*Phaseolus vulgaris* L.	#	#	C	Se	Half beans are applied on the skin affected by a dog bite; when the beans fall off, the wound is healed	+	
Fabaceae							
*Plantago lanceolata* L.	**Bar prenash, Dell, Lulë dheli, Premie**	**Жилавец**	W	L	Crushed and topically applied on wounds: haemostatic	+	+
Plantaginaceae							
CAME 26284							
*Plantago major* L.							
CAME 26261							
Plantaginaceae							
					Infusion: for treating stomachaches		+
*Primula veris* L.	**Lulë aguliçe, Lula dasht, Sgarifet**	**Гороцвеке**	W	FAP	Infusion: panacea, cough		+
Primulaceae							
CAME 26317							
					Infusion: intestinal troubles in kids		+
					Infusion: externally applied on eye inflammations		+
*Prunus domestica* L.	#	#	C	Fr	Fermented and distilled in raki, topically applied, especially for wounds	+	+
Rosaceae							
CAME 26239							
					Fermented and distilled into raki, which is drunk hot with sugar for treating cold		+
					Fermented and distilled into raki, externally applied with salt for treating toothache		+
*Prunus spinosa* L.		#	W	Fr	Infusion: anti-rheumatic and anti-fever		+
Rosaceae							
CAME 26260							
					Infusion: stomachache anti-diarrheal	+	
*Pyrus pyraster* (L.) Burgsd.	#	#	W	Fr	Decoction of the dried fruits with sugar: stomachaches		+
Rosaceae							
CAME 26244							
*Pyrus amygdaliformis* Vill.							
Rosaceae							
CAME 26316							

*Ribes multiflorum* Kit. ex Roem. et Schult.		#	W	Fr	Consumed as snack and for treating digestive discomfort		+
Grossulariaceae							
CAME 26263							
*Rosa canina* L. s.l.	**Karametha, Kroc, Kroza**	**Шипинка**	W	Fr	Infusion: panacea	+	+
Rosaceae							
CAME 26237							
					Infusion: anti-diarrheal, stomachaches		+
					Infusion: sore throats, bechic, flu	+	+
					Infusion: to treat “seven diseases”, blood depurative, diuretic, cardiotonic, anti-fever		+
*Rubus hirtus* Waldst. et Kit.	#	#	W	Fr	Oleolite in topical application: anti-haemorrhoidal	+	
Rosaceae							
CAME 26258							
*Rubus ulmifolius* Schott							
Rosaceae							
CAME 26310							
*Rubus caesius* L.							
Rosaceae							
CAME 26245							
					Fermented and distilled into raki, which is considered cardiotonic		+
				L	Infusion: for treating stomachaches, anti-diarrheal, esp. in children	+	
				Sh	Externally applied on skin for treating infections	+	
*Sambucus ebulus* L.		Див боз	W	Fr	Externally for treating herpes		+
Adoxaceae							
CAME 26254							
*Sideritis raeseri* Boiss. et Heldr.	Çai i bardhë, Çai mali		W	FAP	Infusion: flu	+	
Lamiaceae							
CAME 26281							
*Solanum tuberosum* L.	#	#	C	T	Externally applied (in slices) for treating eye inflammations or head-aches*		+
Solanaceae							
*Taraxacum officinale* Weber ex F.H. Wigg.	#	#	W	Fl	As part of the *lule ditvere* (“flower of the summer”) bunch, which is hung on the churn, as a good omen for the diary production		+
Asteraceae							
CAME 26289							
*Thymus longicaulis* C. Presl.		Полски чаj	W	AP	Infusion: panacea		+
Lamiaceae							
CAME 26272							
*Tilia platyphyllos* Scop.	**Çaj blini**	**Пушала**	W	Fl	Infusion: panacea	+	+
Malvaceae							
CAME 26241							
					Infusion: recreational, flu		+
*Ulmus minor* Mill. and other *Ulmus* spp.	Vidh		W	G	Infusion: anti-hepatitis	+	
Ulmaceae							
CAME 26303							
*Urtica dioica* L.	#	#	W	L	Externally rubbed on the affected part (eventually with salt): anti-rheumatic	+	
Urticaceae							
CAME 26262							
				L; R	Infusion: anti-rheumatic	+	+
				YL	Cooked with rice with rice, eggs, and dairy products (*buranje*^ALB^*/zelje*^MAC^), as post-partum reconstituent		+
				AP	Externally applied for treating bruises	+	+
					Externally rubbed on breasts in cows affected by the evil-eye		+
				R	Decoction: anti-rheumatic	+	
*Vaccinium myrtillus* L.	#	#	W	Fr	Snack for treating stomachache		+
Ericaceae							
					Infusion: anti-fever		+
*Verbascum longifolium* Ten.		Допушке	W	L	Infusion: flu		+
Scrophulariaceae							
CAME 26287							
Diverse tree species			W; C	WC	Hot charcoal put in water and the resulting liquid in external washes on the face of the child suffering from the evil-eye; or thrown on the person suspected to be the gazer		+
				WC	Powdered and applied on the mom’s breast to wean the baby*	+	
Diverse tree species			W; C	DW	Smoked, as a deterrent for bees and then anti-bites		+
				WA	Externally applied (warm) on the cheek for treating toothache or on the neck for treating tonsillitis		+
Not identified		Млечка	W	AP	Infusion: hepatitis		+

^§^: first record of the species in Albania.

In bold: folk taxa quoted by more than 40% of the informants.

C: cultivated; SD: semi-domesticated; W: wild.

*: past use.

#: see Table 1.

+: recorded use.

Plant part(s) used: *AP* aerial parts; *Bu* bulbs; *DFB* dried fruiting body; *DW* decayed wood; *FAP* flowering aerial parts; *FB* flowering branches; *Fl* flowers; *Fr* fruits; *FT* flowering tops; *G* galls; *J* juice; *L* leaves; *R* roots; *Se* seeds; *Sh* Shoots; *T* tubers; *Th* thorns; *UF* unripe fruits; *WA* ashes from wood; *WC* charcoal from wood; *YL* young leaves.

### Veterinary plants

The uses of 57 plant taxa for ethnoveterinary purposes are reported in Table 3. Apart from a certain number of fodder plants and a few medicinal remedies, a large portion of this section of the local ethnobotany is represented by plants that are used ritually for the *Georgyovden* feast (corresponding to St. George’s Day), in order to propitiate good health for the animals or a successful season for the dairy products. This tradition is especially relevant within the Macedonian community and it is well rooted within other South Slavic customs. In Bulgaria, for example, the St. George’s Day is associated with plant decorations being used to “protect” the animals and the house: *Salix* spp., *Juglans regia*, *Artemisia* spp., *Clematis vitalba*, *Glechoma hederacea*, *Veronica officinalis*, *Chamaecytisus hirsutus*, *Convallaria majalis*, *Ranunculus acris*, *Caltha palustris*, *Ajuga* spp., *Lamium purpureum,* and *Ranunculus ficaria*[65,67,68].

**Table 3:** Local plants considered for improving the animals’ well-being in the study area

**Taxon, family and voucher specimen code**	**Folk name(s) recorded among Albanians**	**Folk name(s) recorded among Macedonians**	**Status**	**Plant part(s) used**	**Recorded local use(s)/perceptions(s)**	**Alb**	**Mac**
*Acer campestre* L.		Клен	W	Br	Fodder (goats)		+
Sapindaceae							
CAME 26252							
*Acer pseudoplatanus* L.		Jавор	W	Br	Fodder		+
Sapindaceae							
CAME 26313							
*Achillea millefolium* L.	=	=	W	FAP	Infusion: for treating rumination troubles		+
Asteraceae							
CAME 26294							
*Allium sativum* L.	#	#	C	Bu	In necklaces to be worn on the cow’s horns against the evil-eye (*sysh, naok*); evil-eye symptoms include the animal not producing milk	+	+
Amaryllidaceae							
					Crushed, mixed with salt, and given as fodder to the sheep on St. George’s Day (May 6^th^): considered propitiatory for the good health of the animals	+	
*Alnus glutinosa* (L.) Gaertn.		Габор	W	Br	Fodder		+
Betulaceae							
CAME 26307							
*Arctium minus* (Hill) Bernh.	Kokuta		W	AP	Fodder	+	
Asteraceae							
CAME 26296							
*Arctium lappa* L.							
Asteraceae							
*Avena sativa* L.		Овес	C	Fr	Fodder, esp. considered good for the horse’s coat		+
Poaceae							
*Beta vulgaris* L.	#	#	C	L	Fodder (raw or in decoctions)	+	+
Amaranthaceae							
*Bovista* sp.	=	=	W	DFB	Externally applied on wounds as an haemostatic (horses)		+
Agaricaceae							
*Capsicum annuum* L.	#	#	C	Fr	Lacto-fermented; the resulting fruits opened and externally applied on the forehead for treating headaches		+
Solanaceae							
*Carpinus orientalis* Mill.	Shkoza	Шкоз	W	Br	Fodder at St. George’s Day (considered as a good omen)		+
Betulaceae							
CAME 26301							
*Chelidonium majus* L.	Gjak edhe qumësht		W	AP	Crushed, mixed with salt, and given as fodder to the sheep on St. George’s Day (May 6th): considered propitiatory for the good health of the animals, but also as a blood depurative and galactagogue	+	
Papaveraceae							
CAME 26250							
*Chenopodium album* L.	Llabot		W	AP	Fodder	+	
Amaranthaceae							
CAME 26300							
*Clematis vitalba* L.	Kurpna	Повит	W	AP	Fodder	+	
Ranunculaceae							
CAME 26259							
							
*Cornus mas* L.	#	#	W	Fl	Honey plant	+	
Cornaceae							
CAME 26279							
*Corylus avellana* L.	#	#	W	Fl	Honey plant	+	
Betulaceae							
CAME 26242							
				Br	Fodder (sheep and goats)		+
*Crataegus monogyna* Jacq.	#	#	W	Fl	Honey plant		+
Rosaceae							
CAME 26280							
*Crataegus sericea* Dzekov^§^							
Rosaceae							
CAME 26278							
				FB	Hung on churns and stable doors on St. George’s Day (May 6^th^) as a good omen for the dairy production		+
*Cruciata laevipes* Opiz	=		W	AP	Crushed, mixed with salt, and given as fodder to the sheep on St. George’s Day (May 6^th^): considered propitiatory for the good health of the animals	+	
Rubiaceae							
CAME 26276							
*Cucurbita maxima* Duch.	#	#	C	Fr	Fodder		+
Cucurbitaceae							
*Cydonia oblonga* Mill.	#	#	C	Fl	Honey plant	+	
Rosaceae							
CAME 26290							
				L	Infusion: stomachache		
				FB	Hung on churns and stable doors on St. George’s Day (May 6^th^) as a good omen for the dairy production		+
*Euphorbia characias* L.	#		W	WP	Considered poisonous and irritating the skin	+	
Euphorbiaceae							
CAME 26283							
*Fagus sylvatica* L.	#	#	W	Fr	Fodder (esp. sheep)	+	+
Fagaceae							
CAME 26249							
				L	Fodder, esp. for sheep and equines	+	+
				Wo	Burned, as repellent for the bees when removing honey from the hives		
*Fraxinus excelsior* L.	=		W	Br	Fodder for sheep		+
Oleaceae							
CAME 26304							
							
*Helleborus odorus* Waldst. et Kit. ex Willd.	=	=	W	AP	Ritually hung on doors and gates on March 13^th^ as a good omen	+	+
Ranunculaceae							
CAME 26282							
				R	Inserted on the horse ear: panacea	+	
				BFAP	Hung on the entry gates (to homes and stables), or on the churn on St. George's Day (May 6^th^): considered a good omen	+	+
*Helichrysum plicatum* DC. and other *Helichrysum* spp.	=	=	W	FAP	Infusion: for treating rumination troubles and diarrhea; kerato-conjunctivitis in sheep		+
Asteraceae							
CAME 26274							
*Hordeum vulgare* L.		Jачмен	C	Fr	Fodder, esp. considered good for improving the beauty of horse’s coat		+
Poaceae							
*Malus domestica* Borkh.	#	#	C	L	Fodder for goats		+
Rosaceae							
CAME 26236							
				FB	Hung on churns and stable doors on St. George’s Day (May 6^th^) as a good omen for the dairy production		+
*Medicago sativa* L.	Jonxha Njonxhë		C	AP	Fodder; considered good for improving the beauty of horse’s coat	+	+
Fabaceae							
CAME 26292							
				AP	Galactagogue for animals	+	
*Melissa officinalis* L.	=	=	W	Fl	Honey plant	+	
Lamiaceae							
CAME 26235							
*Populus nigra* L.	Plepi		W	L	Fodder	+	
Salicaceae							
CAME 26302							
*Primula veris* L.	=	=	W	FAP	Hung on churns and stable doors on St. George’s Day (May 6^th^) as a good omen		+
Primulaceae							
CAME 26317							
*Prunus avium* (L.) L.	#	#	W	Br	Fodder		+
Rosaceae							
CAME 26240							
*Prunus domestica* L.	#	#	C	L	Fodder for goats		+
Rosaceae							
CAME 26239							
*Prunus cerasus* L.	#	#	C	Br	Fodder		+
Rosaceae							
CAME 26298							
*Pteridium aquilinum* (L.) Kuhn	Fier		W	L	Bedding for animals	+	
Dennstaedtiaceae							
CAME 26315							
*Quercus cerris* L.	**Bung, Çarri, Dushk, Lis**	**Добк**	W	Fr	Fodder for sheep and goats	+	+
Fagaceae							
CAME 26256							
*Quercus frainetto* Ten.							
Fagaceae							
CAME 26246							
							
				Br	Dried, and stored in loft: fodder	+	+
			W	Sa	Externally instilled in the ear for treating earaches		+
*Robinia pseudoacacia* L.	Akac, Bagren		W	Fl	Honey plant	+	
Fabaceae							
CAME 26305							
*Rosa canina* L. s.l.			W	Fl	Honey plant		+
Rosaceae							
CAME 26237							
*Salix alba* L.	Shelçë, Shelgë		W	L	Fodder for goats	+	
CAME 26251							
Salicaceae							
*Salix eleagnos* Scop.							
Salicaceae							
CAME 26248							
*Salix purpurea* L.							
Salicaceae							
CAMNE 26255							
			W	Fl	Honey plant	+	
*Salvia verticillata* L.	**Gombelik, Lule bulli**	**Гомбели**	W	AP	Fodder	+	+
Lamiaceae							
*Sambucus ebulus* L.	=	=	W	Fr	Externally for treating wounds in sheep		+
Adoxaceae							
CAME 26254							
					Consumed by cats and dogs on their own when they do not feel well	+	
				Fr	Fermented and distilled into *raki* (rare)	+	
*Secale cereale* L.	#	#	C	St	Galactagogue for animals (esp. given to the cows one month before giving birth)	+	+
Poaceae							
*Solanum tuberosum* L.	#	#	C	T	Fodder	+	
Solanaceae							
*Syringa vulgaris* L.		Jоргован	C	FB	Hung on churns and stable doors on St. George’s Day (May 6^th^) as a good omen for the dairy production		+
Oleaceae							
CAME 26309							
*Tanacetum macrophyllum* (Waldst. et Kit.) Sch. Bip.		Вратика	W	AP	Together with nettles, this is rubbed on the goat’s mammaries on St. George’s day (May 6^th^) to improve milk production		+
Asteraceae							
CAME 26269							
*Tanacetum vulgare* L.			W	AP	Hung on churns and stable doors on St. George’s Day (May 6^th^) as a good omen for the dairy production		+
Asteraceae							
CAME 26268							
					Mixed with salt and given to sheep who are thirsty		+
					Fodder		+
*Taraxacum officinale* Weber ex F.H. Wigg.	#	#	W	Fl	Crushed, mixed with salt, and ritually given as fodder to the animals on St. George’s Day (May 6^th^): considered a good omen and galactagogue		+
Asteraceae							
CAME 26289							
					Honey plant		+
*Trifolium pratense* L.	Detelina		W	L	Fodder for sheep	+	+
Fabaceae							
CAME 26297,							
*Trifolium incarnatum* L. ssp. *molineri* (Hornem.) Ces.							
Fabaceae							
CAME 26318, and other *Trifolium* spp.							
Fabaceae							
					Honey plant		+
*Urtica dioica* L.	#	#	W	AP	Rubbed onto the mammaries of cows affected by the Evil-Eye		+
Urticaceae							
CAME 26262							
					Together with *Tanacetum macrophyllum*, this is rubbed onto goat mammaries on St. George’s day (May 6^th^) to improve milk production		+
					Hung on churns and stable doors on St. George’s Day (May 6^th^) as a good omen for dairy production		+
					Fodder		+
*Vicia ervilia* (L.) Willd.		Уров	C	Se	Fodder		+
Fabaceae							
*Zea mays* L.	#	#	C	Fr	Fodder, esp. for increasing the growth speed of lambs and for improving the coat of horses	+	+
Poaceae							
					Galactagogue for all animals		+
Diverse tree species			W; C	WA	Repellent against other insects in the bee hives		+
Not identified		Лула манушаче	W	FAP	Hung on home gates, churns and stable doors on St. George’s Day (May 6^th^) as a good omen		+
Not identified	Spenger		W	R	Inserted on the animal ear for treating diverse diseases	+	

^§^: first record of the species in Albania.

In bold: folk taxa quoted by more than 40% of the informants.

C: cultivated; SD: semi-domesticated; W: wild.

*: past use.

#: see Table 1.

=: see Table 2.

+: recorded use.

Plant part(s) used: *AP* aerial parts; *Br* branches; *Bu* bulbs; *BAFP* Branches with flowering aerial parts; *FAP* flowering aerial parts; *FB* flowering branches; *Fl* flowers; *Fr* fruits; *L* leaves; *R* roots; *Sa* sap; *Se* seeds; *St* stems; *T* tubers; *Wo* wood; *WA* ashes from wood; *WP* whole plant.

Drazheva has analyzed the coincidence of St. Georges’ Day with the most important spring feast in rural Bulgaria, which is widespread with varied rituals [69]. According to this review, one of the main circles connected with St. George's Day focuses around the ritual taking of the sheep to their summer pasture, the ritual milking, the sacrificial practices devoted to a saint who has inherited the characteristic features of the patron-ancestor of the Thracian Heroes, including the open-air feast usually associated with them. A second circle of rites and customs connected to *Georgyovden* is intended for guaranteeing health and well-being for the family, with fortune-telling about the forthcoming wedding feasts for the young people, which is directly related with the reproduction of the community in both its biological and social dimensions.

### Cross-cultural ethnobotany: Macedonian vs. Albanian plant knowledge

From our analysis of the overlap between the Macedonian and Albanian ethnobotanies, we could point out that majority of plant reports (approx. half) were quoted by Macedonians only. However, this could be due to the uneven sample selection between the two field studies: the number of the Macedonian informants was roughly three times larger than the number of the Albanian interviewees. On the other hand, it is important to note that only extremely limited new information was found in both communities after the first dozen in-depth interviews. Our findings could support the persistence of a more “herbophilic” attitude among South-Slavs, as we have already postulated in previous cross-cultural comparative studies in the Western Balkans [26,46]. Moreover, since Macedonians were and also are those in the study area who trade/sell the largest share of wild crafted medicinal herbs to the nearby Albanian towns (i.e. Elbasan, Tirana) and markets or via Albanian middle men, their knowledge of these plants remains within their sphere of household economics. Thus, these activities may have delayed the decrease of local plant knowledge among this population.

The plant reports found in common between the two communities are approx. one third of the overall recorded plant reports (Jaccard Index: 0.29). This would demonstrate some diverging trajectories of the ethnobotanies of the two groups, despite many years of living together in the same area and sharing the same religious faith. Nevertheless, these commonalities demonstrate how cultural edges are particularly significant in bio-cultural diversity [70].

**Figure 4:**
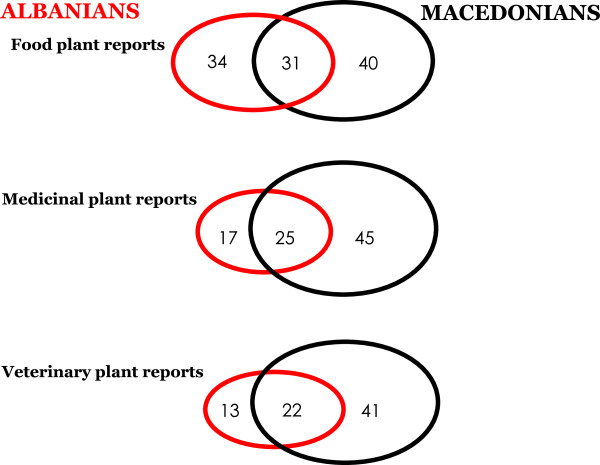
Diagram representing the overlaps between the food, medicinal, and veterinary plant reports recorded among Macedonians and Albanians in the study area.

The overlaps of the folk plant reports in the three considered domains (food, human medicine, and veterinary) are represented in Figure 4. In all three domains, the Jaccard Index measuring the similarity of the data sets collected among Albanians and Macedonians in Gollobordo is 0.29, although internal uses of medicinal plants (teas) and also ritual uses of veterinary plants made at *Georgyovden* seem to be much more relevant among Macedonians.

We recently applied the concept of resilience to migrants’ ethnobotanies [45], while defining resilience as the capability of socio-ecological systems to absorb disturbances and to retain their basic structures and functions, which includes in particular four pillars [71]: 1) the capability of the systems of learning to live with change and absorb it; 2) of nurturing diversity for reorganisation and renewal; 3) of combining different kinds of knowledge for learning; and 4) of creating opportunities for self-organisation. The remarkable resilience evident in the Macedonian medical and veterinary ethnobotanies is indicative of a complex cultural adaptation processes that this community underwent. Moreover, the isolation of this community may related to the difficulties that Macedonians have experienced in accessing the mainstream Albanian culture and institutionalized health as well; the proof of this isolation can be seen in the generation of elderly women, who are the health care givers within the households and often still show difficulties in fluently speaking the Albanian language. Isolation could be ultimately seen then also as a kind of adaptive mechanism. This also shows how negotiations among diverse ethnic groups in mountainous areas could be linked to the practice of symbiotic relations and pluralism, as in the case studies of the Wakhi and Kyrgyz and Pashtu and Shugni of the Pamir [72,73].

In 1956, Fredrik Barth proposed for his field site in the mountains of Swat, North Pakistan, a path-breaking reflection for those times concerning the link between the use of certain ecological niches and ethnic boundaries [74]. According to his observations, the distribution of ethnic groups ecological niches is controlled by the distribution of species each group is able to exploit. Moreover, different ethnic groups may exploit the same ecological niche only if the weaker of them would be better in using marginal environments. The history of Gollobordo’s Macedonians in the last century seems to confirm this, since this group remained concentrated in the highest and more inhospitable village sites, while Albanians began to replace Macedonians in the villages located to lowest altitudes. The Macedonian group had to learn to make use of these marginal areas and the affiliated local resources, including the use of potato leaves for food and the reliance on several herbal medicines, in both the domestic arena and for trade. Although partly symbiotic, the relationship between the two communities has not been equal and the Macedonians have occupied the more marginalized socio-cultural niche.

## Conclusions

Local environmental resources derived from plants continue to play an important role in the provision of dietary and medical care for both humans and their livestock in Gollobordo’s communities. We could confirm a more *herbophilic* attitude of the Macedonians, especially with regards to medicinal and veterinary plants, while the overlaps between the Albanian and the Macedonian ethnobotanies are still relatively limited (restricted to a quarter of the overall recorded plant reports). This confirms that in Gollobordo, despite the two communities having shared the same religion and the same environmental space for many decades, the “original” TEK systems still persist, perhaps due to the geographical and cultural isolation of the area, especially with regards to the Macedonian community. Initiatives aimed at generating an endogenous rural development and especially at fostering sustainable gathering activities of local plants – as well as their small-scale trade and eco-tourism – should seriously consider these cultural divergences. This could in turn promote a tighter collaboration between the two communities and help to sustain the threatened linguistic and cultural heritage of the Macedonian minority.

## Competing interest

The authors declare that they have no competing interest.

## Authors’ contribution

AP and KV conducted the field study and identified the plant samples. AP, AN, and CLQ analyzed the collected data; AH and BM contributed to the comparison with the Albanian ethnobotanical data; AN conducted the comparison with the Bulgarian ethnobotany and folklore; AP and CLQ wrote the manuscript and drafted the discussion. All authors read and approved the final manuscript.

## References

[B1] Łuczaj Ł Zovkokoncic M Milicevic T Dolina K Pandza M Wild vegetable mixes sold in the markets of Dalmatia (southern Croatia) J Ethnobiol Ethnomed 2013 9 2 10.1186/1746-4269-9-2 23286393 PMC3554486

[B2] Łuczaj Ł Nieroda Z Collecting and learning to identify edible fungi in southeastern Poland: age and gender differences Ecol Food Nutr 2011 50 4 319 336 10.1080/03670244.2011.586314 21888599

[B3] Papp N Birkás-Frendl K Farkas A Pieroni A An ethnobotanical study on home gardens in a Transylvanian Hungarian Csángó village (Romania) Genet Resour Crop Evol 2013 60 1423 1432 10.1007/s10722-012-9930-7

[B4] Pieroni A Quave CL Giusti ME Papp N “We are Italians!”: the hybrid ethnobotany of a Venetian diaspora in Eastern Romania Hum Ecol 2012 40 435 451 10.1007/s10745-012-9493-4

[B5] Mustafa B Hajdari A Pajazita Q Syla B Quave CL Pieroni A An ethnobotanical survey of the Gollak region, Kosovo Genet Resour Crop Evol 2011 59 1 16

[B6] Mustafa B Hajdari A Krasniqi F Hoxha E Ademi H Quave CL Pieroni A Medical ethnobotany of the Albanian Alps in Kosovo J Ethnobiol Ethnomed 2012 8 6 10.1186/1746-4269-8-6 22284581 PMC3285519

[B7] Šarić-Kundalić B Dobeš C Klatte-Asselmeyer V Saukel J Ethnobotanical survey of traditionally used plants in human therapy of east, north and north-east Bosnia and Herzegovina J Ethnopharmacol 2011 133 3 1051 1076 10.1016/j.jep.2010.11.033 21094241

[B8] Šarić-Kundalić B Dobeš C Klatte-Asselmeyer V Saukel J Ethnobotanical study on medicinal use of wild and cultivated plants in middle, south and west Bosnia and Herzegovina J Ethnopharmacol 2010 131 1 33 55 10.1016/j.jep.2010.05.061 20594943

[B9] Redzic S Wild medicinal plants and their usage in traditional human therapy (Southern Bosnia and Herzegovina, W. Balkan) J Med Plants Res 2010 4 11 1003 1027

[B10] Redzic S Barudanovic S Pilipovic S Wild Mushrooms and Lichens used as Human Food for Survival in War Conditions; Podrinje - Zepa Region (Bosnia and Herzegovina, W. Balkan) Research in Human Ecology 2010 17 2 175 187

[B11] Redžić S The ecological approach to ethnobotany and ehnopharmacology of population in Bosnia and Herzegovina Collegium Antropol 2007 31 869 890 18041402

[B12] Redžić S Wild edible plants and their traditional use in the human nutrition in Bosnia and Herzegovina Ecol Food Nutr 2006 45 189 232 10.1080/03670240600648963

[B13] Savikin K Zdunic G Menkovic N Zivkovic J Cujic N Terescenko M Bigovic D Ethnobotanical study on traditional use of medicinal plants in South-Western Serbia, Zlatibor district J Ethnopharmacol 2013 146 3 803 810 10.1016/j.jep.2013.02.006 23422337

[B14] Jarić S Popović Z Mačukanović-Jocić M Djurdjević L Mijatović M Karadžić B Mitrović M Pavlović P An ethnobotanical study on the usage of wild medicinal herbs from Kopaonik Mountain (Central Serbia) J Ethnopharmacol 2007 111 1 160 175 10.1016/j.jep.2006.11.007 17145148

[B15] Menković N Šavikin K Tasić S Zdunić G Stešević D Milosavljević S Vincek D Ethnobotanical study on traditional uses of wild medicinal plants in Prokletije Mountains (Montenegro) J Ethnopharmacol 2011 133 1 97 107 10.1016/j.jep.2010.09.008 20837123

[B16] Pieroni A Local plant resources in the ethnobotany of Theth, a village in the Northern Albanian Alps Genet Resour Crop Evol 2008 55 8 1197 1214 10.1007/s10722-008-9320-3

[B17] Pieroni A Pardo de Santayana M, Pieroni A, Puri RK People and plants in Lëpushë. Traditional medicine, local foods, and post- communism in a North Albanian village Ethnobotany in the new Europe: People, health and wild plant resources 2010 New York/Oxford: Berghahn 16 50

[B18] Babai D Molnár Z Multidimensionality and scale in a landscape ethnoecological partitioning of a mountainous landscape (Gyimes, Eastern Carpathians, Romania) J Ethnobiol Ethnomed 2013 9 1 10.1186/1746-4269-9-1 23388111 PMC3610200

[B19] Dénes A Papp N Babai D Czúcz B Molnár Z Wild plants used for food by Hungarian ethnic groups living in the Carpathian Basin Acta Soc Bot Pol 2012 81 4 381 396 10.5586/asbp.2012.040

[B20] Molnár Z Classification of pasture habitats by Hungarian herders in a steppe landscape (Hungary) J Ethnobiol Ethnomed 2012 8 28 10.1186/1746-4269-8-28 22853549 PMC3533854

[B21] Nedelcheva A Dogan Y Usage of plants for weather and climate forecasting in Bulgarian folk traditions Indian J Tradit Knowl 2011 10 1 91 95

[B22] Nedelcheva A Dogan Y Obratov-Petkovic D Padure IM The traditional use of plants for handicrafts in southeastern Europe Hum Ecol 2011 39 6 813 828 10.1007/s10745-011-9432-9

[B23] Dogan Y Nedelcheva AM Obratov-Petković D Padure IM Plants used in traditional handicrafts in several Balkan countries Indian J Tradit Knowl 2008 7 1 157 161

[B24] Papp N Bartha S Boris G Balogh L Traditional uses of medicinal plants for respiratory diseases in Transylvania Nat Prod Commun 2011 6 10 1459 1460 22164782

[B25] Kołodziejska-Degórska I Mental herbals - A context-sensitive way of looking at local ethnobotanical knowledge: Examples from Bukovina (Romania) Trames 2012 16 3 287 301 10.3176/tr.2012.3.04

[B26] Rexhepi B Mustafa B Hajdari A Rushidi-Rexhepi J Quave CL Pieroni A Traditional medicinal plant knowledge among Albanians, Macedonians and Gorani in the Sharr Mountains (Republic of Macedonia) Genet Resour Crop Evol 2013 60 2055 2080 10.1007/s10722-013-9974-3

[B27] Zlatković BK Bogosavljević SS Radivojević AR Pavlović MA Traditional use of the native medicinal plant resource of Mt. Rtanj (Eastern Serbia): Ethnobotanical evaluation and comparison J Ethnopharmacol 2014 151 1 704 713 10.1016/j.jep.2013.11.037 24296087

[B28] Łuczaj Ł Fressel N Perković S Wild food plants used in the villages of the Lake Vrana Nature Park (northern Dalmatia, Croatia) Acta Soc Bot Pol 2013 82 4 275 281 10.5586/asbp.2013.036

[B29] Berkes F Sacred Ecology: Traditional Ecological Knowledge and Resource Management 1999 Philadelphia: Taylor & Francis

[B30] Kathe W Honnef S Heym A Medicinal and aromatic plants in Albania, Bosnia-Herzegovina, Bulgaria, Croatia and Romania 2003 Bonn, Germany: BfN

[B31] Londoño PT Doka D Becker H Collection of medicinal and aromatic plants in Albania - An analysis given by examples of the surroundings of Peshkopi (Dibër Region) Zeitschrift fur Arznei- und Gewurzpflanzen 2008 13 4 153 160

[B32] Łuczaj Ł Pieroni A Tardío J Pardo-De-Santayana M Sõukand R Svanberg I Kalle R Wild food plant use in 21st century Europe: The disappearance of old traditions and the search for new cuisines involving wild edibles Acta Soc Bot Pol 2012 81 4 359 370 10.5586/asbp.2012.031

[B33] Hadjichambis A Paraskeva-Hadjichambi D Della A Giusti ME De Pasquale C Lenzarini C Censorii E Gonzales-Tejero MR Sanchez-Rojas CP Ramiro-Gutierrez JM Skoula M Sarpaki A Hmamouchi M Jorhi S El-Demerdash M El-Zayat M Pieroni A Wild and semi-domesticated food plant consumption in seven circum-Mediterranean areas Int J Food Sci Nutr 2008 59 5 383 414 10.1080/09637480701566495 18979618

[B34] Karousou R Balta M Hanlidou E Kokkini S "Mints", smells and traditional uses in Thessaloniki (Greece) and other Mediterranean countries J Ethnopharmacol 2007 109 2 248 257 10.1016/j.jep.2006.07.022 16962274

[B35] Tomićević J Bjedov I Obratov-Petković D Milovanović M Exploring the park-people relation: Collection of *vaccinium myrtillus* L. by local people from Kopaonik National Park in Serbia Environ Manage 2011 48 4 835 846 10.1007/s00267-011-9725-1 21800263

[B36] Bigaran F Mazzola A Stefani A Enhancing territorial capital for developing mountain areas: The example of Trentino and its use of medicinal and aromatic plants Acta Geographica Slovenica 2013 53 SPL.2

[B37] Zuin MC Lante A Zocca F Zanin G Zanin G A phytoalimurgic garden to promote wild edible plants Acta Horticulturae 2010 881 855 858

[B38] Luczaj L Kohler P Piroznikow E Graniszewska M Pieroni A Gervasi T Wild edible plants of Belarus: from Rostafinski's questionnaire of 1883 to the present J Ethnobiol Ethnomed 2013 9 21 10.1186/1746-4269-9-21 23557012 PMC3627636

[B39] Pieroni A Rexhepi B Nedelcheva A Mustafa B Hajdari A Kolosova V Cianfaglione K Quave CL One century later: the folk botanical knowledge of the last remaining Albanians of the upper Reka Valley, Mount Korab, Western Macedonia J Ethnobiol Ethnomed 2013 9 22 10.1186/1746-4269-9-22 23578063 PMC3648429

[B40] Łuczaj Ł Changes in the utilization of wild green vegetables in Poland since the 19th century: a comparison of four ethnobotanical surveys J Ethnopharmacol 2010 128 2 395 404 10.1016/j.jep.2010.01.038 20097282

[B41] Kalle R Sõukand R Wild plants eaten in childhood: A retrospective of Estonia in the 1970s-1990s Bot J Linean Soc 2013 172 2 239 253 10.1111/boj.12051

[B42] Kalle R Sõukand R Historical ethnobotanical review of wild edible plants of Estonia (1770 s-1960s) Acta Soc Bot Pol 2012 81 4 271 281 10.5586/asbp.2012.033

[B43] Soukand R Kalle R Change in medical plant use in Estonian ethnomedicine: a historical comparison between 1888 and 1994 J Ethnopharmacol 2011 135 2 251 260 10.1016/j.jep.2011.02.030 21376110

[B44] Sõukand R Kalle R Where does the border lie: Locally grown plants used for making tea for recreation and/or healing, 1970s-1990s Estonia J Ethnopharmacol 2013 150 162 174 10.1016/j.jep.2013.08.031 23994468

[B45] Ceuterick M Vandebroek I Pieroni A Resilience of Andean urban ethnobotanies: a comparison of medicinal plant use among Bolivian and Peruvian migrants in the United Kingdom and in their countries of origin J Ethnopharmacol 2011 136 1 27 54 10.1016/j.jep.2011.03.038 21470576

[B46] Pieroni A Giusti ME Quave CL Cross-cultural ethnobiology in the Western Balkans: Medical ethnobotany and ethnozoology among Albanians and Serbs in the Pešter Plateau, Sandžak, South-Western Serbia Hum Ecol 2011 39 3 333 349 10.1007/s10745-011-9401-3

[B47] Łuczaj Ł Archival data on wild food plants used in Poland in 1948 J Ethnobiol Ethnomed 2008 4 4 10.1186/1746-4269-4-4 18218132 PMC2275233

[B48] Albanian Institute of Statistics (INSTAT) Gross Domestic Product for Republic of Albania [http://www.instat.gov.al/media/101280/llogarite_rajonale_4faqeshi_ang_.pdf]

[B49] Steinke K Yilli X Die slavische Minderheiten in Albanien 2007 Munich: Verlag Otto Sagner

[B50] Sobolev AN Novik AA Golo Bordo (Gollobordë), Albanija. Iz materialov balkanckoj ekspedicii RAN i SPbGU 2008–2010 gg. [Golo Bordo (Gollobordë), Albania. From the material of the Balkan expedition of the Saint Petersburg State University 2008–2010] 2013 Verlag Otto Sagner: Munich

[B51] International Society of Ethnobiology Statement on Ethics: Principles of Professional Responsability [http://www.aaanet.org/profdev/ethics/upload/Statement-on-Ethics-Principles-of-Professional-Responsibility.pdf]

[B52] Qosia X Paparisto K Demiri M Vangjeli J Balza E Flora e Shqipërisë 2 1992 Tirana: Akademia e Shkencave e Republikes se Shqipërisë, Instituti i Kërkimeve Biologjike

[B53] Paparisto K Demiri M Mitrushi I Qosia X Flora e Shqipërisë 1 1988 Tiana: Akademia e Shkencave e RPS të Shqipërisë, Qendra e Kërkimeve Biologjike

[B54] Qosia X Paparisto K Vangjeli J Babi R Flora e Shqipërisë 3 1996 Tirana: Akademia e Shkencave e Republikes se Shqipërisë, Instituti i Kërkimeve Biologjike

[B55] Vangjeli J Ruci B Mullaj A Paparisto K Qosia X Flora e Shqipërisë 4 2000 Tirana: Akademia e Shkencave e Republikes se Shqipërisë, Instituti i Kërkimeve Biologjike

[B56] Demiri M Flora Ekskursioniste e Shiperise 1983 Shtëpia Botuese e Librit Shkollor: Tirana

[B57] Euro-Med Euro+Med PlantBase. The information resource for Euro-Mediterranean plant diversity http://ww2.bgbm.org/EuroPlusMed/PTaxonDetail.asp?NameId=7713605&PTRefFk=7300000

[B58] Stevens PF Angiosperm Phylogeny Website 2012

[B59] Stoianovich T Le maïs dans le Balkans Annales Économies, Sociétes, Civilisations 1966 5 1026 1040

[B60] Andrews J Diffusion of Mesoamerican food complex to Southeastern Europe Geogr Rev 1993 83 194 204 10.2307/215257

[B61] Galaty ML Lafe O Lee WE Light and Shadow: Isolation and Interaction in the Shala Valley of Northern Albania 2013 Los Angeles, USA: Cotsen Institute of Archaeology

[B62] Brown MS McDonald GM Friedman M Sampling leaves of young potato (*Solanum tuberosum*) plants for glycoalkaloid analysis J Agric Food Chem 1999 47 6 2331 2334 10.1021/jf981124m 10794631

[B63] Johns T With bitter herbs they shall eat it. Chemical ecology and the origins of human diet and medicine 1990 Tucson, USA: University of Tucson Press

[B64] Ahtarov B Davidov B Yavashev A Materiali za Balgarski botanichen rechnik [Materials for the Bulgarian botanical glossary] 1939 Sofia: Balgarska Akademia na Naukite, Pridvorna Pechatnitsa

[B65] Georgiev M Balgarska narodna medicina. Enciklopedia [Bulgarian folk medicine. Encyclopedia] 2013 Akademichno Izdatelstvo Prof. Marin Drinov: Sofia

[B66] Almaleh M Ezikat na tsvetovete. Tsvetovete v Balkanskiya folklor [The language of color. The colours of the Balkan folklore] 2007 Izdatelstvo Askoni: Sofia

[B67] Marinov D Izbrani proizvedenija. 1.2 Religiozni narodni obichai [Selected works. 1.2 Religious folk customs] 2003 Iztok-Zapad: Sofia

[B68] Marinov D Izbrani proizvedenija. 1.1 Narodne vyara [Selected works. 1.1 Folk beliefs] 2003 Iztok-Zapad: Sofia

[B69] Drazheva R Georgyov Den Ethnologia Bulgarica 1990 2 3 13

[B70] Turner NJ Davidson-Hunt IJ O'Flaherty M Living on the edge: Ecological and cultural edges as sources of diversity for social-ecological resilience Hum Ecol 2003 31 3 439 462 10.1023/A:1025023906459

[B71] Folke C Colding J Berkes F Berkes F, Colding J, Folke C Synthesis: building resilience and adaptive capacity in social-ecological systems Navigating Social-Ecological Systems Building Resilience for Complexity and Change 2003 Cambridge: Cambridge University Press 352 387

[B72] Kassam KA Lozny LR Keeping all the parts: Adaptation amidst dramatic changes in the Pamir mountains Continuity and Change in Cultural Adaptation to Mountain Environments 2013 New York: Springer 303 317

[B73] Kassam KAS Pluralism, resilience, and the ecology of survival: Case studies from the Pamir Mountains of Afghanistan Ecology and Society 2010 15 2 9

[B74] Barth F Ecological relationships of ethnic groups in Swat, North Pakistan Am Anthropol 1956 58 1079 1089 10.1525/aa.1956.58.6.02a00080

